# Attitude Towards Suicide among Caregivers of Patients Admitted with Suicide Attempt in a Tertiary Care Hospital: A Descriptive Crosssectional Study

**DOI:** 10.31729/jnma.6246

**Published:** 2021-04-30

**Authors:** Prekshya Thapa, Sami Lama, Nirmala Pradhan, Kriti Thapa, Rajesh Kumar, Madhur Basnet

**Affiliations:** 1 Department of Psychiatric Nursing, College of Nursing, B. P. Koirala Institute of Health Sciences, Dharan, Nepal; 2 Department of Psychiatry, B. P. Koirala Institute of Health Sciences, Dharan, Nepal

**Keywords:** *attitude*, *caregivers*, *suicide*, *Nepal*

## Abstract

**Introduction:**

Understanding the attitude of caregivers towards suicide attempters could be useful in suicide prevention. The objective of this study was to study attitude towards suicide among caregivers of patients with suicide attempt admitted to a tertiary care hospital in Nepal.

**Methods:**

A descriptive cross-sectional study was conducted with 52 caregivers of patients with suicide attempt who had been admitted to a tertiary care hospital of Nepal after obtaining ethical clearance from Institutional Review Committee (ref. IRC/0797/016). Data were collected through interviews using the Attitude towards Suicide Questionnaire and in-depth interviews conducted on five caregivers using the interview framework developed in the department for the purpose. Data and descriptive analysis were done using Statistical Package for the Social Sciences version 21. Point estimate at 95% Confidence Interval was calculated along with frequency and percentage for binary data. Content analysis was used for qualitative data.

**Results:**

Majority of the caregivers 34 (65.4%) had unfavorable attitude towards suicide. Caregivers reported that mental and chronic physical illness, financial difficulty, working environment, and social factors such as undue pressure and failure to perform the task, difficulty maintaining the relationship, abuse, and neglect could be some of the possible causes of suicide. Caring and understanding attitude of family members, health professionals, and society towards the suicidal individuals providing appropriate training and education to the public would help in reducing the stigma and burden of suicidal patients.

**Conclusions:**

The overall attitude of the caregivers was unfavorable. Interventions targeted towards improving attitude towards suicide could be helpful in suicide prevention.

## INTRODUCTION

Suicide is defined as “an act of self-destruction, initiated and committed by a person fully aware of the fatal outcome” and represents a major public health problem worldwide.^1,2^ Nepal had an estimated 24.9 suicides per 100,000 people in 2012.^3,4^

Caregivers play a vital role in suicide prevention by providing physical, psychological, spiritual, and emotional supports.^5^ Attitude affects emotions, cognition, and behavior and thus the caregiver's attitude towards suicide patients can affect the quality of care the suicidal patients receive.^6,7,8^ While attitudes towards suicide have been studied over the past decade in diverse groups,^9^ limited data on caregiver's attitudes towards suicide are available.^10, 11^

The objective of this study was to study attitude towards suicide among caregivers of patients with suicide attempt admitted to a tertiary care hospital in Nepal.

## METHODS

This descriptive cross-sectional was conducted to find out attitude towards suicide among caregivers of patients admitted with suicidal attempts. Quantitative and qualitative data were collected at the same time and the methodology was primarily quantitative. The study setting was B. P. Koirala Institute of Health Sciences (BPKIHS), a Health Sciences University located in Dharan. The study was carried out between December 2016 and June 2017 after obtaining ethical clearance from the Institutional Review Committee of BPKIHS (IRC/0797/016). Data collection was done from 1st to 31st March 2017 using convenience sampling. There were 57 caregivers of patients with suicidal attempts during the enrollment period who met the eligibility criteria and consented to participate in the study and all of them were enrolled in the study.

$$\begin{array}{ll} \text{n} & \text{= }{\text{Z}}^{\text{2}}\times (\text{p}\times \text{q)}/{\text{e}}^{\text{2}}\\ & \text{= }1.96^{\text{2}}\times 0.5\times (1-0.5)/\text{0}{\text{.13}}^{\text{2}}\\ & \text{= }57 \end{array}$$

where,

n = required sample size
p = prevalence of unfavourable attitude, 50%
q = 1-p
e = margin of error, 13%
Z = 1.96 at 95% Confidence Interval

Five of them were used for pretest and they were excluded from the final analysis. Five of the caregivers of patients with suicidal attempts admitted inwards were taken for an in-depth interview. All caregivers who were above 18 years of age and caring for the patients with suicidal attempts admitted in various wards of BPKIHS and who were referred to the psychiatry department for evaluation were eligible for participation. Only those caretakers who had been with the patient continuously for at least six months and had been taking the primary responsibility of the patient were considered as the caregivers for the study. Those caregivers who were diagnosed with mental or other chronic illness were excluded.

The procedure and purpose of the study were explained to the participants and informed consent was read to the caregivers and once the consent was signed, a face-to-face interview was conducted. The qualitative data were collected using an interview schedule guide in the respective wards or psychiatry OPD in separate rooms maintaining confidentiality and comfort by the lead author herself. Attitude Towards Suicide (ATTS) scale; a 5 point Likert scale was used to assess the attitude towards suicide.^12^ We used items 4 to 40 of the ATTS in our study. Each item has five options-strongly agree (1), agree (2), undecided (3), disagree (4), and strongly disagree(5), with the least total sum score of 37 to the highest of 185. Items 7 and 9 are reverse scored.

A higher score implies a negative attitude. There is no definite cutoff point in previous studies. As this is a five-point Likert Scale with score three being the mean score, we took the cutoff point of 60% of the total score to identify favorable or unfavorable attitude. Attitude score >60% was taken as unfavorable and <60% was taken as a favorable score. For an in-depth interview, the following two open-ended questions were asked to the caregivers.

1. What do you think is the main reason why people commit suicide? 2. What do you think should be done to prevent suicide?

Ethical clearance was obtained from the Institutional Review Committee of BPKIHS. Informed written consent was obtained from each of the participants before enrolling in the study. All the points of good clinical practice and ethical research were duly followed throughout the study.

After collection of data, they were checked for completeness, organized, coded, and entered into Microsoft Excel, and converted into Statistical Package for Social Sciences 21.0 version. Descriptive statistics (frequency, percentage) were calculated. For qualitative analysis, all interviews were audio-taped and transcribed verbatim. Each written interview transcript was examined for statements; major statements were extracted from the descriptions and meanings were formulated from the significant statements. The formulated meanings were sorted and then organized into categories, theme clusters, and themes to identify the experiences common to all participants with consensus among all the investigators.

The study was conducted in a tertiary care setting among caregivers of suicide attempting patients who needed hospitalization and during the acute care period. During the period of such an acute state attitude towards suicidal patients might be negative. So the findings might not be generalizable to other populations of caregivers. However, we have tried to minimize this influence by enrolling the cases after the acute care had been taken care of and patients were medically stable. This is a descriptive cross-sectional study and so we could only see the association between various factors and attitude and further studies are needed to find the cause-effect relationship.

The study was supported with NRs. 20,000/- grant support from the university.

## RESULTS

Most of the caregivers 35 (66.6%) as well as patients 34 (65.4%) were Hindu by religion. Homemaking was the predominant occupation among both caregivers 17 (32.7%) and patients 14 (26.9%). Siblings 18 (34.6%), parents 14 (26.9), and spouse 9 (17.3%) were the main caregivers. The majority of caregivers 30 (57.7%) as well as patients 31 (59.6%) were from joint family. The majority of 46 (88.5%) of the patients had attempted once. The majority of 41 (78.8%) of the caregivers had a hospital stay less than 5 days (Table 1).

**Table 1:** Sociodemographic characteristics of the caregivers and patients (n = 52).

Characteristics	Category	n (%)	
		Caregivers	Patients
Age(in years)	Upto 20years	7 (13.5)	16 (30.8)
	21-40 years	26 (50.0)	28 (53.8)
	>40 years	19 (36.5)	8 (15.4)
Mean age (SD)		36.0 (11.23)	28.2(12.3)
	Male	27 (51.9)	23 (44.2)
Gender	Female	25 (48.1)	28 (53.8)
	Others	0 (0)	1 (1.9)
	Married	45 (87)	30 (57.7)
Marital status	Unmarried	7 (13)	16 (30.8)
	Divorced	0	5 (9.6)
	Widow/widower	0	1 (1.9)
Education level	Literate	47 (90.4)	43 (82.7)
	Illiterate	5 (9.6)	9 (17.3)
Mode of suicide attempt	Self-Poisoning		43 (82.7)
	Hanging		6(1.5)
	Others		3 (5.8)

**Caregiver's Attitude towards Suicide**

**Figure 1.**
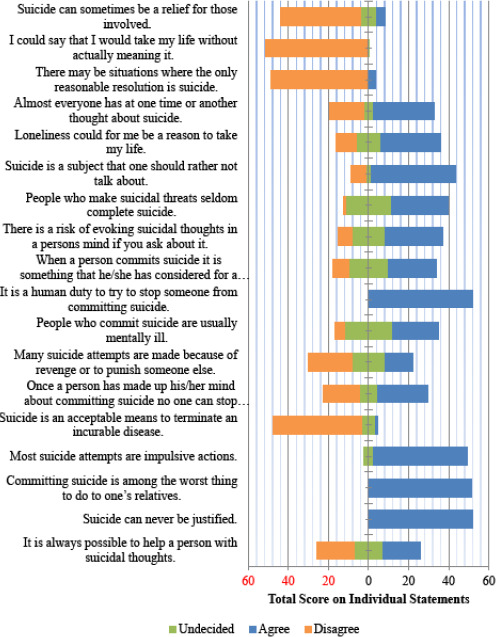
Attitude towards suicide (ATTS) score on individual statements (1-18).

**Figure 2.**
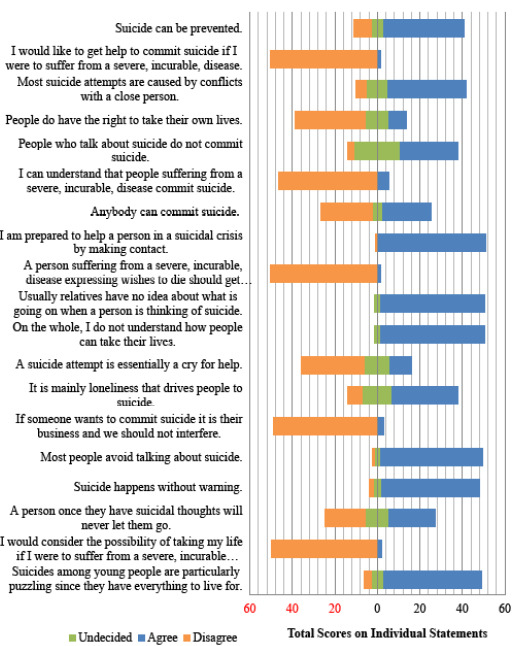
Attitude towards Suicide (ATTS) Score on individual statements (19-37).

The majority of 34 (65.4%) of the caregivers had an unfavorable attitudes towards suicide. The caregiver's attitude towards suicide on individual statements of the ATTS is shown in Figures 1 and 2. As the number of participants is less, we collapsed the five options of ATTS into three as- “strongly disagree” and “disagree” into disagree, “strongly agree” and “agree” into agree and undecided for ease of data display. Some of the major findings on various domains were as follows:

**Opinion on suicide as a right:**

There was a favorable attitude on the “suicide as a right” domain: “Suicide can never be justified.” 52 (100% agreed); “If someone wants to commit suicide it is their business and we should not interfere.” 49 (94.2% disagreed) (Figures 1 and 2).

**Understanding of suicide:**

There was an unfavorable attitude among the majority of the participants in this domain which showed that the participants had a relatively poor understanding regarding issues related to suicide. Some of the major statements with unfavorable attitude were: Majority 49 (94.2%) of the caregivers agreed on the statement “Usually relatives have no idea about what is going on when a person is thinking of suicide.” “Suicides among young people are particularly puzzling since they have everything to live for.” 46 (88.5% agreed); “Most suicide attempts are impulsive actions.” 47 (90.4% agreed) and “Suicide happens without warning.” 46 (88.5% agreed) (Figures 1 and 2).

**Prevention of suicide:**

There was a favorable attitude towards the majority of the statements on “prevention of suicide” domain as: “It is a human duty to try to stop someone from committing suicide” 52 (100%); “I am prepared to help a person in a suicidal crisis by making contact.” 51 (98.1%); “Suicide can be prevented,” 36 (73.1%). The majority 42 (80.8%) of the caregivers agreed on “Suicide is a subject that one should rather not talk about.”

Results of in-depth interview: The following were the major theme identified from the in-depth interview (Table 2).

**Table 2:** Major themes were identified from the indepth interview.

Question	What do you think is the main reason why people commit suicide?	What do you think should be done to prevent suicide?
Themes Identified	Mental and Chronic physical Illness	Caring and understanding Attitude towards suicidal individuals
	Financial unemployment status leading to stress	Raising public awareness and the need for additional professional assistance
	Working environment	
	Social Factors: Undue family pressure and failure to perform the task Difficulty maintaining relationship/ marriage Abuse/Neglect	

**1. What do you think is the main reason why people commit suicide?**

Mental illness/Chronic physical illness: The caregiver expressed some of the possible causes of suicide as mental illness such as depression ("Now what I understood is that it happened because of depression only."-ID No.1) and chronic illness ("I have seen ill people also committing suicide. In those people with chronic illnesses, family members can't afford to treat them and in such situation also people do so.” - ID No.2) ("For him, he had the idea that my illness won't be cured, I would die. So, he did so."- IDNo.3)

Financial and employment status: Another major cause reported was financial difficulty ("It could be because of poor financial status as well. It could also be because of not being able to afford for the study of children because of unemployment.” - IDNo.3); ("Some people do so because of financial condition as well."- IDNo.4)

Working Environment: Almost all of the caregivers expressed a stressful work environment as a possible reason for suicide. Torture at the workplace by senior and colleagues ("I have seen people doing so because of torture at the job, senior's torture at the job."- IDNo.1), inadequate and inappropriate placement ("People study but get no job and go abroad and there is no value of the certificate."- IDNo.2), loss of job ("It can happen if one's job is gone.” - IDNo.3) and stressful working environment ("Now during the job itself, there are a lot of people who give stress sending people here and there."- IDNo.5) as contributing factors for suicide.

Social Factors: There were various important subthemes in the social factors themes which are as follows:

Undue Pressure and Failure to perform Task: Almost all of the caregivers expressed the family expectations for performing better at study ("And the other reason is pressure from family to study."- ID No.1), unhealthy comparison, and inappropriate feedback ("Despite that she gave licensing exams of pharmacy but failed four times. She was scolded for that also- your friend passed but you never study and she did this.” - IDNo.2), procrastination and low self-esteem ("Someone might commit suicide feeling I studied this much but could not do anything. And some may commit suicide while studying itself saying I could not get this many marks, I could not do anything.” - IDNo.3) and failure in examinations ("Some do so if they fail in School Leaving Examinations (SLC). - ID No.4) as possible social factors for suicide.

Difficulty maintaining relationship/Marriage: Caregivers expressed problems in maintaining relationships ("I have seen young boys doing so because they failed in a love affair.” - IDNo.1), being uncared for by spouse ("Son-in-law also stopped caring her, would not care even whether she had taken meals or not. So she did so thinking my life is this much only, it's better to die, they will also get relief."-IDNo.2), or betrayal from a spouse ("It could happen if husband also betrays. If husband listens to others only and does not listen to wife also it can happen."- IDNo.5) as some of the major reasons for suicide.

Abuse/Neglect: Caregivers expressed abuse/neglect as one of the major reasons for suicide. Children being neglected/abused by their parents ("I know such cases where little children have been physically abused and have done so.- IDNo.1), people being sexually abused (Being abused by relatives and neighbors can also lead to such event. Even older people could be raped, marital rape also could occur.” - IDNo.1) or elders being neglected by their family members (". Even when kept at home they are beaten, neglected, not given meals on time and behaved like servants at home.” - IDNo.2; “Offsprings giving torture, not giving meals, not doing treatments on time, not giving them good clothes could be the reasons."- IDNo.3) could be some of the reasons for suicide.

**2. What do you think should be done to prevent suicide?**

Caring and understanding attitude: Most of the caregivers expressed the caring attitude of parents and family members towards their children ("Family members should love their children, understand their wishes and know what makes them happy. They have to be behaved, spoken to and be understood like friends.” - IDNo.1; “Parents as well as the neighbors have the responsibility to guide them in the right direction by providing them a good environment and understanding what they are wishing to do, wear, study.” IDNo.2) understanding attitude towards suicidal persons ("We have to understand the matters of one who has tried to commit suicide and address that. Like, help if any financial problem, help if we know anything, help if they are ill and other things like that.” IDNo.3) and a helpful attitude towards suicidal people ("We have to give love and care, let no scarcity in anything, give them appropriate advice when they wish to do something to prevent suicide.” IDNo.4) could help prevent suicide.

Raising public awareness and the need for additional professional assistance: Another major theme identified regarding suicide prevention was raising public awareness and the need for professional assistance. They expressed that public and caretaker awareness and health education ("For prevention of suicide, first we have to raise awareness, we have to take education related to that to the households in the villages. And for attendants like us, there should be training regarding health education.” IDNo.1) awareness about mental illnesses and availability of health professionals ("There is need to spread public awareness regarding mental illness. There should be such doctors in the health post who is well educated, well educated about mental illness and able to give good counseling.” IDNo.2) and role of health care workers in raising awareness ("Health workers like you have to convince him that this suicide attempt of mine is a mistake, I should not die, now I need to get well.” IDNo.5) could play a vital role in suicide prevention.

## DISCUSSION

The current study showed that the majority of the caregivers have an unfavorable attitude towards suicide. This may be because suicide carries a tremendous stigma in Nepalese society.^13,14^ However, these findings contradict the study conducted by Chiang et.al.^7^ The caregivers reported mental illness, chronic physical illness, working environment, social factors, and stress due to financial status are some of the major factors for suicide which resonates with the findings of other studies.^15,16^ The caregivers also expressed that the caring and understanding attitude of family members, society, and health professionals could help in suicide prevention. Likewise, caregivers expressed raising public awareness on mental illness and the need for professional assistance for the prevention of suicide which was similar to other studies.^17,18^

All of the participants had agreed (44% strongly agree and 56% agree) that it is a human duty to try to stop someone from committing suicide. This was reflected during the qualitative interviews as well. All the participants expressed that every individual, family members as well as society have the responsibility to take care of someone who is suicidal and help him/her. All the participants had responded that suicide can never be justified (52% strongly agree, 48% agree). During the in-depth interview also, the participants agreed that there could be various reasons behind one's suicide but no reason is an acceptable justification for suicide. Similarly, most of them had responded that most people avoid talking about suicide (23% strongly agree and 69% agree). It was reflected during the in-depth interview as well. The participants responded that they often avoid talking about suicide by their relatives because of the stigma and negative views regarding suicide prevalent in society. One of the participants responded- “I did not tell about my daughter even to a close relative of mine whose wife had delivered in this hospital only and I had gone to visit them as well because we have a negative attitude about it here.” Overall, the findings of the in-depth interview complemented the findings of the quantitative study and it also helped us to get further insights into the caregivers' experiences of caregiving.

## CONCLUSIONS

These study findings revealed that the overall attitude towards suicide was unfavorable. The attitude was unfavorable mainly in the “understanding about suicide” domain. It was found that the caring and understanding attitude of family members, health professionals, and society would help in promoting a positive environment among the suicidal patients and thus help in decreasing suicidal rates. Raising awareness and providing appropriate training and education to the public as well as health personnel would help in reducing the stigma and burden of suicidal patients.
